# Rapamycin improves social and stereotypic behavior abnormalities induced by pre‐mitotic neuronal subset specific *Pten* deletion

**DOI:** 10.1111/gbb.12854

**Published:** 2023-06-28

**Authors:** David A. Narvaiz, Suzanne O. Nolan, Gregory D. Smith, Andrew J. Holley, Conner D. Reynolds, Katherine J. Blandin, Phuoc H. Nguyen, Doan L. K. Tran, Joaquin N. Lugo

**Affiliations:** ^1^ Department of Psychology and Neuroscience Baylor University Waco Texas USA; ^2^ Institute of Biomedical Studies Baylor University Waco Texas USA; ^3^ Department of Biology, Baylor University Waco Texas USA

**Keywords:** ASD, autism, comorbidity, cortical dysplasia, epilepsy, mTOR, PI3K, Ps6, social behavior, stereotypic behavior

## Abstract

The mechanistic target of rapamycin (mTOR) pathway is a signaling system integral to neural growth and migration. In both patients and rodent models, mutations to the phosphatase and tensin homolog gene (*PTEN*) on chromosome 10 results in hyperactivation of the mTOR pathway, as well as seizures, intellectual disabilities and autistic behaviors. Rapamycin, an inhibitor of mTOR, can reverse the epileptic phenotype of neural subset specific *Pten* knockout (NS‐*Pten* KO) mice, but its impact on behavior is not known. To determine the behavioral effects of rapamycin, male and female NS‐*Pten* KO and wildtype (WT) mice were assigned as controls or administered 10 mg/kg of rapamycin for 2 weeks followed by behavioral testing. Rapamycin improved social behavior in both genotypes and stereotypic behaviors in NS‐*Pten* KO mice. Rapamycin treatment resulted in a reduction of several measures of activity in the open field test in both genotypes. Rapamycin did not reverse the reduced anxiety behavior in KO mice. These data show the potential clinical use of mTOR inhibitors by showing its administration can reduce the production of autistic‐like behaviors in NS‐*Pten* KO mice.

## Graphical abstract

Inhibition of the mechanistic target of rapamycin (mTOR) pathway through rapamycin during adolescent improved stereotypy and social behavior in neural subset specific *Pten* (NS‐Pten) knockout mice. Rapamycin reduced locomotor behavior but did not impact anxiety behavior in wildtype mice.

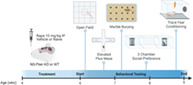

**Abbreviations**
mTOR: mechanistic target of rapamycin
PTEN: phosphatase and tensin homolog deleted on chromosome ten

## 1 INTRODUCTION

Autism spectrum disorder (ASD) is a neurodevelopmental disorder that has been estimated to affect 1 in 54 children in the United States.^1^ ASD presents with deficits in both social communication and restricted, repetitive patterns of behavior that can impair academic and occupational functioning. These impairments are also associated with increased caregiving and healthcare costs^2^ that are markedly concerning given that ASD may disproportionately burden low‐income families.^3^, ^4^ Pharmacological treatments targeting ASD development, however, have yet to be identified.

ASD associated with a single gene or chromosomal defect is highly penetrant and estimated to account for ~5% of individuals diagnosed with ASD.^5^ Many of the pathways involved in monogenic ASD have been identified and are considered promising targets of drug development.^6^, ^7^, ^8^, ^9^ One such pathway is the mechanistic target of rapamycin (mTOR), which is responsible for regulating cell growth and migration in the context of available cellular resources. Mutations to genes of proteins that regulate the mTOR pathway, such as *Tsc1*, *Tsc2* and the phosphatase and tensin homolog (*PTEN*) on chromosome 10, have been linked to the development of ASD.^10^, ^11^, ^12^

Although the discussion of *PTEN* mutations in those with ASD were previously only found in case studies, *PTEN* mutations are now being recognized as a significant contributor in the development of ASD.^11^, ^13^ *PTEN* is a suppressor of the mTOR pathway, important in mediating cell growth and migration during neurodevelopment.^14^ Loss of function mutations to *PTEN* in both humans and rodent models have been associated with ASD and neurological features commonly found comorbid in individuals with ASD, such as macrocephaly and epilepsy.^11^, ^13^, ^15^, ^16^, ^17^ A recent meta‐analysis found that 25% of individuals with *PTEN* mutations were diagnosed with ASD.^11^ ASD‐like behavior, as well as macrocephaly and epilepsy, have also been recapitulated in a variety of *Pten* knockout (KO) models.^15^, ^16^, ^17^ These models, which vary in the spatial, temporal and quantity of *Pten* deletions in the brain, exhibit an assortment of ASD‐like behaviors similar to the heterogeneity of behavioral phenotypes found in humans. In some rodent models, the suppression of hyperactive mTOR signaling induced by PTEN deletion has shown promise in preventing the development of ASD‐like symptoms and commonly found comorbid conditions.^18^, ^19^, ^20^, ^21^ However, because of the heterogeneity of ASD, it is important that these results be repeated in all *Pten* KO models to ensure effective treatments can be identified.

Rapamycin, an allosteric inhibitor of mTOR, can suppress hyperactive mTOR signaling and prevent the development of the autistic‐like phenotype in models where *Pten* deletion occurs after neuronal differentiation.^18^ The neuronal subset specific (NS) *Pten* KO mouse model, however, exhibits *Pten* deletion prior to neuronal differentiation in primarily granule cells of the hippocampus and cerebellum, as well as some pyramidal neurons throughout the cortex.^22^, ^23^ The NS‐*Pten* KO model has been repeatedly found to exhibit deficits in social, repetitive and cognitive behaviors which may represent a more severe phenotype of ASD when compared with other models.^15^, ^24^, ^25^, ^26^, ^27^, ^28^ However, it has not been determined if rapamycin can also prevent the development of ASD‐like behaviors in this model. Here, we determine if a 2‐week course of rapamycin previously shown to suppress seizures in NS‐*Pten* KO mice, can also prevent the development of ASD‐like behavioral deficits.

## 2 MATERIALS AND METHODS

### 2.1 Animals

Mice were neuron subset‐specific *Pten* (NS‐*Pten)* KO mice generated using Cre‐LoxP (GFAP‐Cre; *Pten* ^*loxP/loxP*^) as previously described.^22^, ^23^ NS*‐Pten* ^*loxP/+*^ heterozygote mice were bred to produce NS*‐Pten* ^*+/+*^ wildtype (WT), NS*‐Pten* ^*loxP/+*^ heterozygous (HT) and NS*‐Pten* ^*loxP/loxP*^ KO mice. Only WT and KO mice were used for this study. For the control groups there were 14 female WT and 17 female KO mice, 17 male WT and 14 male KO mice. For the rapamycin treated mice there were 13 female WT and 8 female KO mice, 21 male WT and 13 male KO mice. WT mice were generated from 31 different litters and KO mice from 25 litters. All mice were bred and group housed according to the guidelines set forth by Baylor University's Institutional Care and Use Committee and the National Institute of Health's *Guide for the Care and Use of Laboratory Animals*. Mice were weaned at ~3 weeks of age and kept on a 12 h:12 h diurnal cycle with ad libitum access to food and water.

### 2.2 Treatment

**FIGURE 1:**
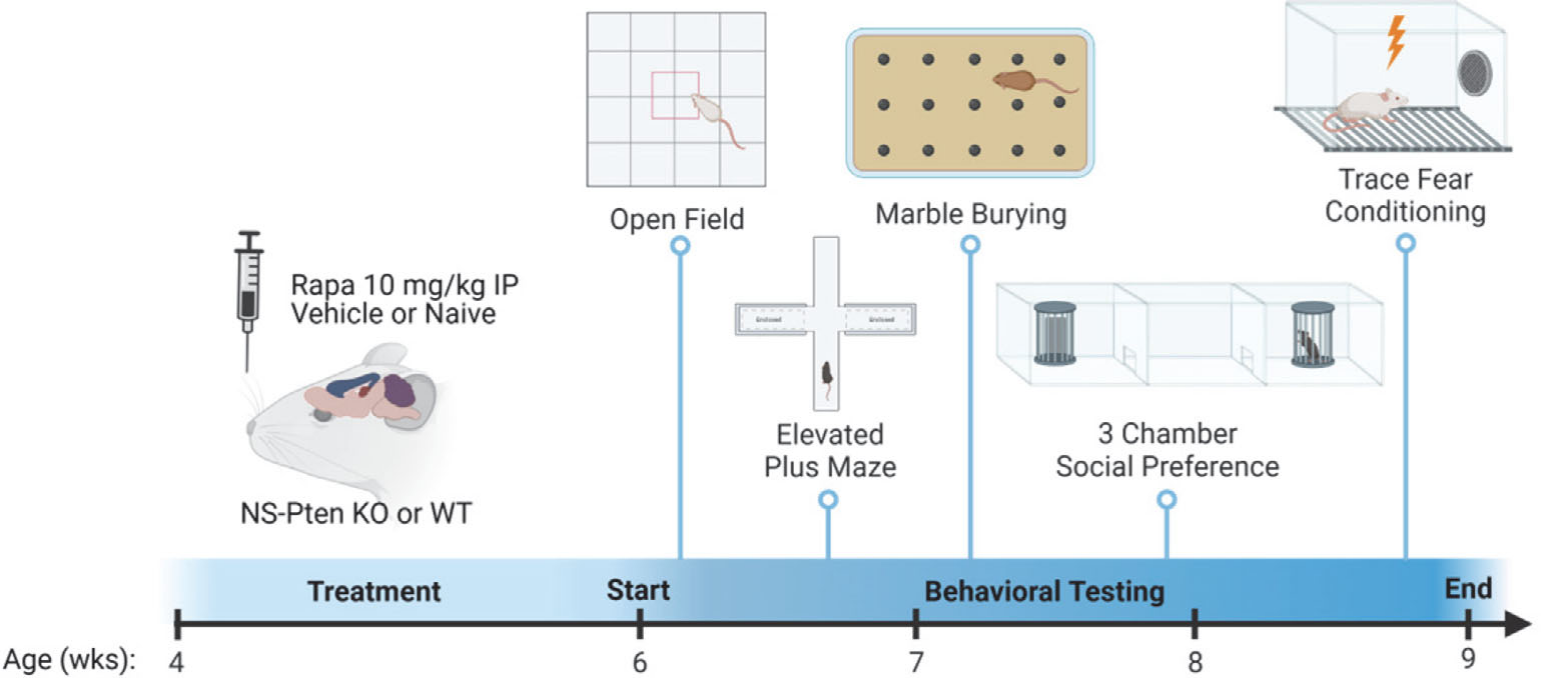
Feeding and behavioral testing timeline. Neural subset specific *Pten* knockout (NS‐*Pten* KO) or wildtype (WT) mice were naïve to treatment or given either an intraperitoneal injection of 10 mg/kg of rapamycin or vehicle 5 days a week for 2 weeks (total of 10 days) at 4 weeks old. Behavioral testing began at 6 weeks old and continued for a total of 3 weeks. Behavioral tests administered were the open field, elevated plus maze, marble burying, three‐chamber social preference and trace fear conditioning.

Rapamycin (LC Laboratories, Woburn, MA, United States) was dissolved in a vehicle solution containing 4% ethanol, 5% polyethylene glycol 400 (Sigma, St. Louis, MO, United States) and 5% Tween 80 (Sigma).^29^ To examine the effects of suppressing hyperactive mTOR activity in NS‐*Pten* KO mice, subjects were administered either rapamycin dissolved in the vehicle solution, the vehicle alone, or were naïve to both the vehicle and treatment.^21^ Rapamycin was administered as a 2‐week loading dose prior to behavioral testing at 10 mg/kg intraperitoneally for 10 days (Monday through Friday for 2 weeks) beginning at ~4 weeks old to male and female, WT and KO mice (Figure 1). Vehicle and naïve treated mice were collapsed to form the control group after an initial analysis determined a lack of differences between these groups across each test.

### 2.3 Behavioral testing

Behavioral testing was conducted during the light phase between 8:00 a.m. and 5:00 p.m. Testing began at ~6 weeks of age and occurred over 3 weeks (Figure 1). No more than two behavioral tests occurred per week. All testing areas were cleaned with 30% isopropyl alcohol between each testing session.

#### 2.3.1 Open field

To determine changes to locomotive, stereotyped and anxiety‐like behaviors, all subjects underwent the open field test.^29^ The testing environment consisted of a Fusion Node acrylic arena (40 cm × 40 cm × 30 cm) (Omnitech Electronics, Columbus, OH, United States). Individual mice were placed in the arena for 30 min. Photobeams at the edge of each zone detected activity within the arena using Fusion Software (Omnitech Electronics). Total distance traveled, rearing frequency and rearing time were analyzed as measures of exploratory activity and locomotive behavior. Circling behavior and time spent grooming (stereotypy time) determined differences in stereotyped behavior. Both the distance traveled, and time spent in the center (inner 50%, 20 cm × 20 cm region) of the open field were compared between groups to evaluate the presence of anxiety‐like and exploratory behavior.

#### 2.3.2 Elevated plus maze

The elevated plus maze was used as an additional measure of anxiety‐like behavior.^30^, ^31^ A plus‐shaped maze was suspended 40 cm above the ground. Two arms were enclosed with acrylic walls and two arms were unenclosed. Each arm was 30 cm × 5 cm and stemmed from a 5 cm × 5 cm central platform. The maze was isolated in a temperature, light and noise‐controlled room. Each subject was placed in the center of the maze and allowed to freely explore the maze for 10 min without an experimenter present. Ethovision XT video tracking software (Noldus, Netherlands) was used to record the time each subject spent in the open and closed arms, as well as track the number of entries into the arms.

#### 2.3.3 Marble burying

As a measure of stereotypic and compulsive behaviors, mice underwent a marble burying task used previously.^15^ Mice were placed in a plastic cage containing a grid shaped pattern of 20 black marbles on 3 cm of bedding. The number of marbles at least 75% buried in the bedding after 30 min were compared between groups.

#### 2.3.4 Social preference

Mice were measured for their preference for social interaction using the three‐chambered social preference task modified to include a novel object.^15^, ^32^ The testing apparatus was a clear acrylic box (24.5 in. × 16.75 in. × 8.75 in.) with three chambers separated by a wall with removable partitions. Testing occurred across two phases. During the baseline phase, an empty wire cup was inverted in each of the side chambers (8 in. × 16.75 in. × 8.75 in.) with a tall plastic cylinder placed above the cup to prevent mice from climbing on the cups. Subjects were placed in the center chamber (7.25 in. × 16.75 in. × 8.75 in.) with the partitions removed and allowed to freely explore all three chambers for 10 min. The testing phase consisted of an age‐ and sex‐matched novel mouse housed within the inverted cup in one side chamber and a novel object (Lego® block) within another inverted cup in the opposite side chamber. The novel mouse was placed in the cup for 1 h for 2 days prior to testing to be habituated to the cup. During each phase, test mice were placed in the central chamber with the partitions removed and allowed to explore the entire testing apparatus freely for 10 min. The novel mouse and object were placed on different sides for each test subject as a counter‐balance measure to prevent side‐bias. The time spent in each chamber was recorded in each phase, as well as the time spent at the cups housing the novel mouse and novel object.

#### 2.3.5 Trace fear conditioning

To determine differences in fear memory, contextual and cued fear‐based learning was analyzed using a trace fear conditioning task and Freeze Frame software (Actimetrics, IL, United States).^33^, ^34^ Testing occurred over 4 days, with 24 h between each session. On day 1, to habituate mice to the testing apparatus and determine baseline responses, mice were placed in the chamber for 12 min without either a conditioned stimulus (CS) or unconditioned stimulus (US). Day 2 consisted of a 260 s baseline period followed by 6 CS (white noise)/US (foot shock) pairings. The white nose (70 dB) was presented for 20 s, followed by an 18 s trace interval separating the CS from the US. At the end of the trace interval a 0.5 mA foot shock was given for 2 s. On day 3, the testing chamber was altered to present an unfamiliar context. The smell, sight, layout, sound and feel of the chamber was changed by adding vanilla extract (Adams Extract, Gonzales, TX, United States) in the tray below the floor, altering the lighting, adding a plexiglass section diagonally inside the chamber, activating a fan in the chamber and placing an opaque section of plexiglass over the grid floor. The bedding in the transfer cage was torn paper towels. Mice were placed in this new context for 580 s. For the first 200 s, neither the CS nor US were presented to determine baseline activity within the chamber. Mice were then presented with four iterations of the CS for 20 s followed by an 80 s inter‐trial interval (ITI). The percentage of time spent freezing during the CS, considered as no movement other than breathing, was used as a measure of fear memory. Higher rates of freezing indicate greater fear memory. On day 4, mice were placed in the original context for 3 min and measured for percent of time spent freezing.

### 2.4 Statistical analysis

Data were analyzed using GraphPad Prism 7 (GraphPad Software, La Jolla, California, United States) and SPSS 24 (IBM Corp., Armonk, New York, United States). Data are mean ± SEM, unless otherwise stated. A three‐way analysis of variance (ANOVA) was used to determine the effects of three dichotomous factors―treatment (control, rapamycin), genotype (WT, KO) and sex (male, female) on the model‐specific, a priori determined behavioral variables. Interactions were investigated using pairwise comparisons with either a Bonferroni adjustment, or Mann–Whitney *U* tests when data violated Levene's test of homogeneity of variance (*p* < 0.05) and were not normally distributed as assessed by boxplot. Repeated measures data complied with Mauchly's test of sphericity (*p* > 0.05). A value of *p* < 0.05 was considered significant for each statistical test. All data are graphed, and only statistically significant interactions and main effects not within an interaction are reported below. The data that support the findings of this study are available from the corresponding author upon reasonable request.

## 3 RESULTS

### 3.1 Open field

**FIGURE 2:**
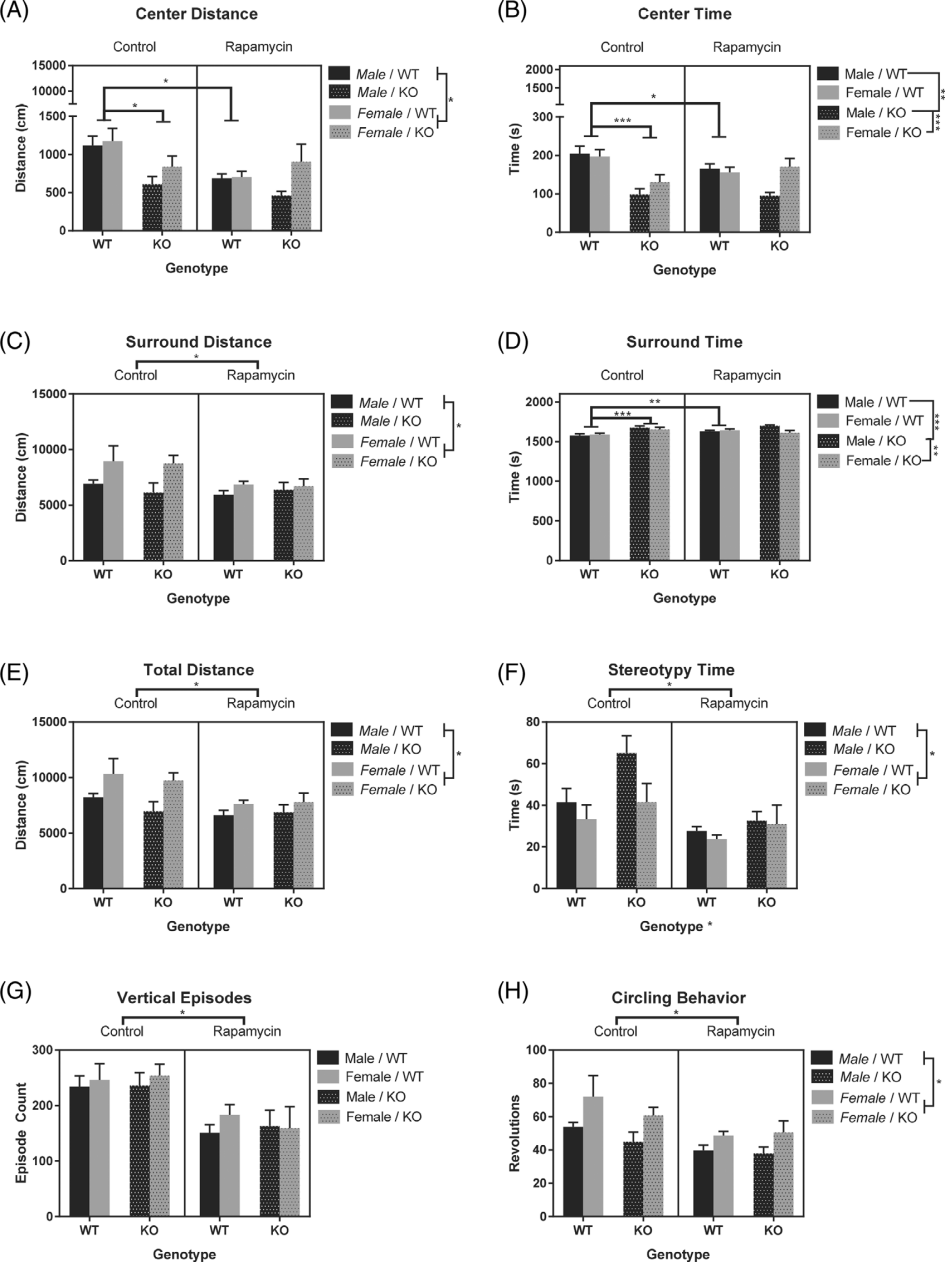
Rapamycin induced a general reduction of activity in the open field. Compared with untreated mice, rapamycin reduced the distance traveled in the center (A) and surround (C), total distance traveled (E), stereotypy time (F), rearing frequency (G) and circling behavior (H). KO mice traveled less in the center compared with controls (A) and spent more time performing stereotypies (F). Wildtype (WT) control mice traveled and spent more time in the center compared with KO controls and WT mice treated with rapamycin (A‐B). Males traveled less distance overall (E) and in the surround (C). Males also performed less circling behavior (H) compared with females. Y axis adjusted for visualization of center distance (A) and center time (B). Data are presented as mean ± SEM. *p < 0.05; **p < 0.01; ***p < 0.001.

Utilizing a three‐way ANOVA, analysis of the distance traveled in the center of the arena showed a main effect of sex, *F*(1, 109) = 4.72, *p* < 0.05, (Figure 2A), with females traveling further than males. There was also a two‐way interaction between treatment and genotype, *F*(1, 109) = 5.69, *p* < 0.05. In the control group, KO mice traveled less distance in the center compared with WT mice, *p* < 0.001. After treatment with rapamycin, there was no difference between WT mice and KO mice in center distance. The effect appears to be primarily on WT mice, as those treated with rapamycin were found to travel less in the center than controls, *p* < 0.001. In KO mice, there was no difference between those treated with or without rapamycin. Analysis of the time in the center showed an interaction between sex and genotype, *F*(1, 109) = 6.90, *p* < 0.05 (Figure 2B). KO females spent more time in the center compared with KO males, *p* < 0.01 and KO males spent less time in the center compared with WT males, *p* < 0.001. There was also an interaction between treatment and genotype, *F*(1, 109) = 6.09, *p* < 0.05 (Figure 2B). WT mice given rapamycin spent less time in the center compared with WT control mice, *p* < 0.05, and WT control mice spent more time in the center than KO control mice, *p* < 0.001. The distance traveled in the surround was investigated and showed a main effect treatment, *F*(1, 109) = 5.47, *p* < 0.05. Control mice were found to travel further than treated mice (Figure 2C). There was also a main effect of sex, *F*(1, 109) = 8.0, *p* < 0.01. Females traveled further than males (Figure 2C). Analysis of the time spent in the surround showed an interaction between sex and genotype, *F*(1, 109) = 6.28, *p* < 0.05 (Figure 2D). KO males spent more time in the surround compared with KO females, *p* < 0.01. Male KO mice also spent more time in the surround compared with male WT mice, *p* < 0.001. There was also an interaction between treatment and genotype, *F*(1, 109) = 5.24, *p* < 0.05 (Figure 2D). WT animals given rapamycin spent more time in the surround compared with WT controls, *p* < 0.01. KO controls also spent more time in the surround compared with WT controls, *p* < 0.001.

The total distance traveled in the open field was analyzed and showed a main effect of treatment, *F*(1, 109) = 8.83, *p* < 0.01, and sex, *F*(1, 109) = 10.15, *p* < 0.01. Rapamycin treated mice traveled less than control mice (Figure 2A), and males traveled less than females (Figure 2E). Stereotypy time was analyzed and showed a main effect of genotype, *F*(1, 109) = 5.78, *p* < 0.05, sex, *F*(1, 109) = 4.09, *p* < 0.05, and treatment, *F*(1, 109) = 12.95, *p* < 0.001 (Figure 2F). KO mice spent more time performing stereotypies compared with WT mice, as did males when compared with females. Treatment with rapamycin induced an overall reduction in stereotypy time. The frequency of rearing events (vertical episodes) was also analyzed, showing a main effect of treatment, *F*(1, 109) = 22.04, *p* < 0.001 (Figure 2G). Mice administered rapamycin performed fewer vertical episodes than control mice. There were no main effects for genotype and no interactions were found. Analysis of circling behavior also showed a main effect of treatment, *F*(1, 109) = 10.05, *p* < 0.01, as well as a main effect of sex, *F*(1, 109) = 10.31, *p* < 0.01 (Figure 2H). Rapamycin reduced circling behavior compared with control mice, and females had higher counts of circling than males. There was no main effect of genotype, and no interactions were found.

### 3.2 Elevated plus maze

**FIGURE 3:**
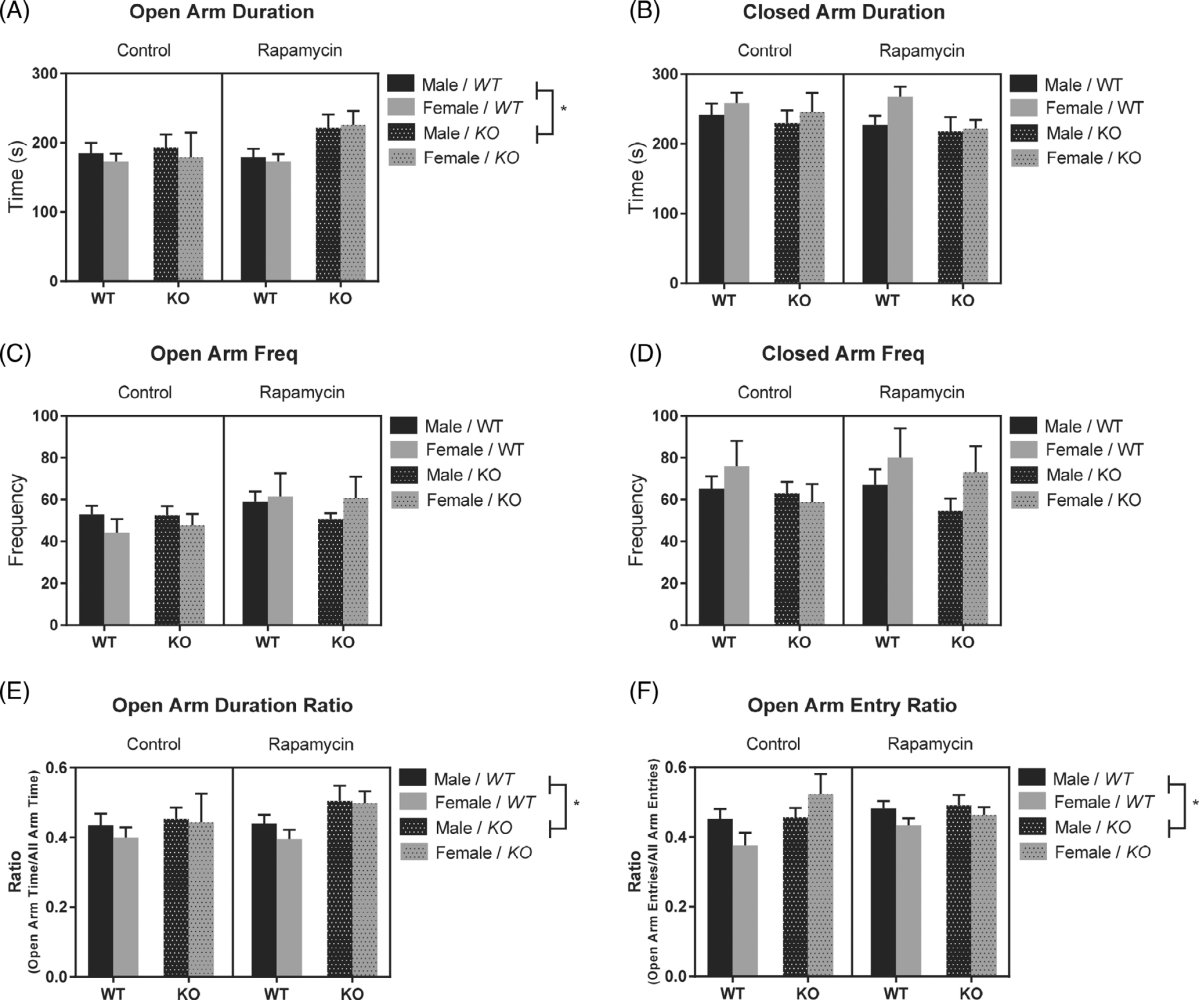
There was no effect of rapamycin in the elevated plus maze. Knockout (KO) mice spent more time in the open arms of the maze compared with wildtype (WT) mice (A). No differences were observed in the time spent in the closed arms (B) or how often subjects entered the open arms (C) or closed arms (D). KO mice also had a greater ratio of time spent (E) and entries into (F) the open arms compared with WT mice. Data are presented as mean ± SEM. *p < 0.05.

Using a three‐way ANOVA we found a significant main effect of genotype on the duration of time spent in the open arms, *F*(1, 94) = 4.55, *p* < 0.05, with KO mice spending more time in the open arms when compared with WT mice (Figure 3A). No differences were found in the amount of time spent in the closed arms (Figure 3B), the number of entries into the open arms (Figure 3C), or into the closed arms (Figure 3D). There was a main effect of genotype in the ratio of time spent in the open arms to time spent in all arms, with KO mice exhibiting a greater ratio of time spent in the open arms compared with WT mice, *F*(1, 95) = 4.00, *p* < 0.05 (Figure 3E). There was a similar main effect of genotype in the ratio of the number of entries in the open arms to entries in all arms, with KO mice having a greater ration of entries into the open arms compared with WT mice, *F*(1, 95) = 4.35, *p* < 0.05 (Figure 3F). No other main effects or interactions were found.

### 3.3 Marble burying

**FIGURE 4:**
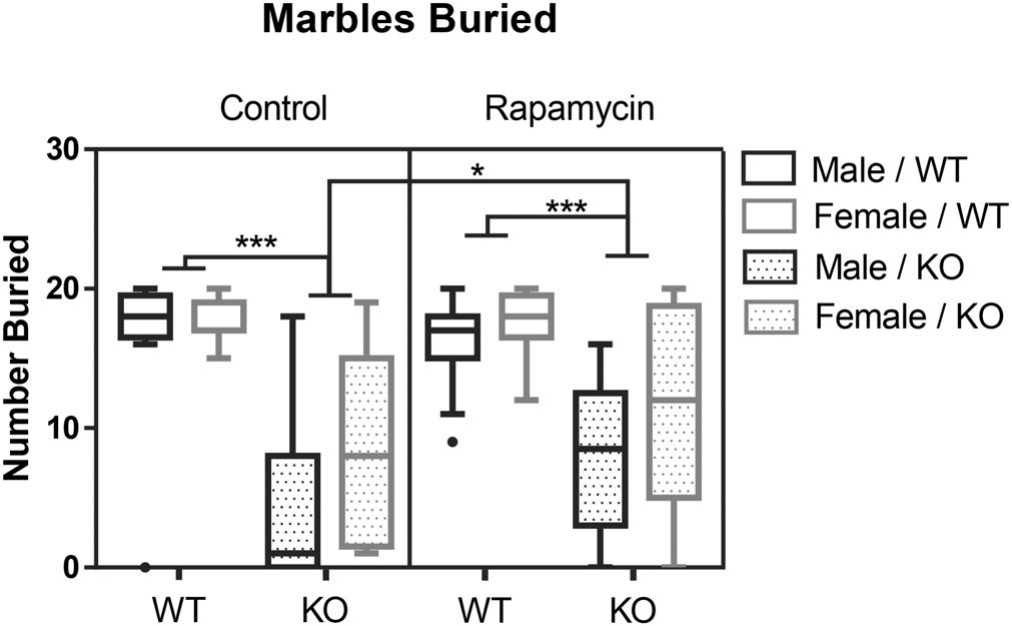
Rapamycin increased marble burying in knockout (KO) mice. KO mice administered rapamycin buried more marbles than control KO mice. Wildtype (WT) control mice buried more marbles than KO control mice. WT rapamycin treated mice buried more marbles than KO rapamycin mice. Data are presented as box‐plot with outliers and median. *p < 0.05; ***p < 0.001.

Using a three‐way ANOVA, the number of marbles buried up to 75% in bedding were analyzed and showed a two‐way interaction between genotype and treatment, *F*(1, 105) = 4.5, *p* < 0.05 (Figure 4). Follow up analyses were performed using Mann–Whitney *U* tests because of violation of Levene's test of homogeneity of variance (*p* < 0.05) and because these groups were not normally distributed as assessed by boxplot. Rapamycin treatment increased the number of marbles buried in KO mice compared with KO control mice, *U* = 518, *z* = −0.34, *p* < 0.05, and had no effect on marble burying in WT mice. WT mice buried more marbles than KO mice treated with rapamycin, *U* = 57, *z* = 4.16, *p* < 0.001, or control KO mice, *U* = 788, *z* = 5.44, *p* < 0.001.

### 3.4 Social preference

**FIGURE 5:**
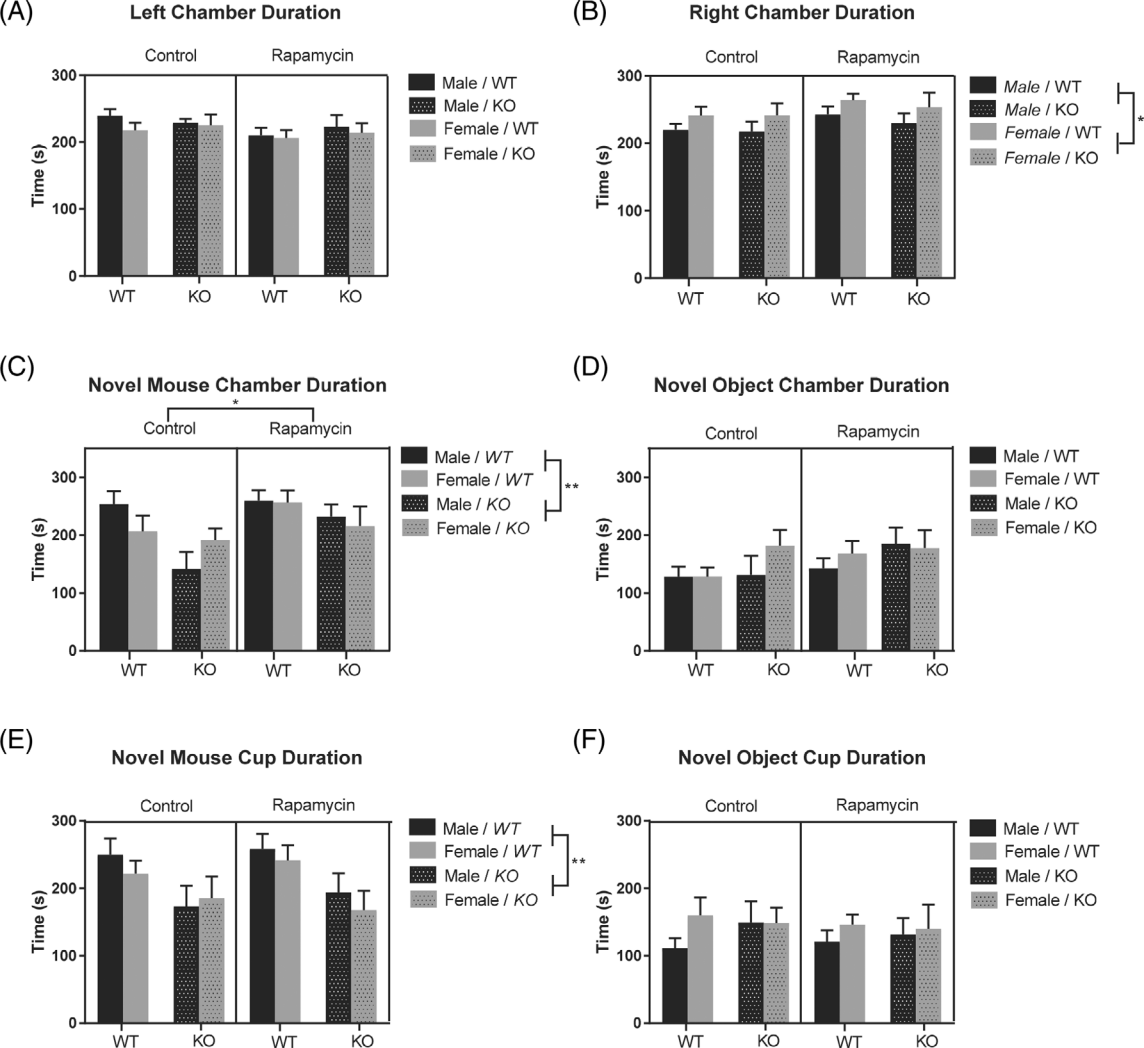
Rapamycin induced a general increase in social preference. During the habituation trial, females spent more time in the right chamber than in the left chamber (A, B). When compared with males, females also spent more time in the right chamber (B). Placement of novel mouse and novel object were thus randomized in subsequent trials to mitigate side bias. During the testing phase, more time was spent in the chamber with the novel mouse than in the chamber containing the novel object by both control and rapamycin treated mice (C, D). Rapamycin treated mice spent more time in the novel mouse chamber than control mice (C). While wildtype (WT) mice preferred the chamber with the novel mouse over the chamber with the novel object, (C, D) there were no differences in knockout (KO) mice (C, D). KO mice also spent less time in the chamber with the novel mouse compared with WT mice (C). Analysis of the time spent at the cups shows the duration of time spent at the novel mouse cup was greater than that of the novel object cup in both KO and WT mice (E, F). However, KO mice spent less time with the novel mouse than WT mice (E). Data are presented as mean ± SEM. *p < 0.05; **p < 0.01; ***p < 0.001.

Five mice were not used because of an equipment malfunction. Two mice were removed for not entering all three chambers, which prevented the determination of preference. After removing these mice, the group sizes were for the control groups: 14 female WT and 16 female KO mice, 16 male WT and 10 male KO mice. For the rapamycin treated mice: 12 female WT and 8 female KO mice, 21 male WT and 13 male KO mice. A three‐way ANOVA was used to determine differences among the three between‐subjects factors (treatment, genotype and sex) on chamber duration. In the first trial of testing, mice were analyzed for differences in time spent in the left and right chambers, each containing an empty wire mesh cup. There were no significant differences in the time spent in the left chamber (Figure 5A). The time spent in the right chamber showed a significant main effect of sex, *F*(1, 98) = 4.98, *p* < 0.05. Females spent more time in the right chamber compared with males (Figure 5B). There were no other significant main effects or interactions.

On the second trial, we placed a novel mouse or novel object under a wire mesh cup in either the left or right chamber. The chambers containing the novel object, or the novel mouse, were randomly assigned to counteract side bias. In the time spent in the chamber housing the novel mouse, a three‐way ANOVA showed a main effect of genotype, *F*(1, 98) = 7.85, *p* < 0.01. KO mice spent less time in the chamber with the novel mouse compared with WT mice (Figure 5C). There was also a main effect of treatment, *F*(1, 98) = 6.08, *p* < 0.05, with mice administered rapamycin spending more time in the chamber with the novel mouse than control mice (Figure 5C). We found no differences in the time spent in the chamber housing the novel object (Figure 5D).

As a further measure of novel mouse interaction, we also assessed the time spent at the cup housing the novel mouse to determine if mice interacted with the novel mouse or merely preferred this chamber. Our analysis showed a main effect of genotype, *F*(1, 98) = 10.85, *p* < 0.01. KO mice spent less time at the cup with the novel mouse than WT mice (Figure 5E). Analysis of the time spent at the cup housing the novel object showed no differences between any of the groups (Figure 5F).

### 3.5 Trace fear conditioning

**FIGURE 6:**
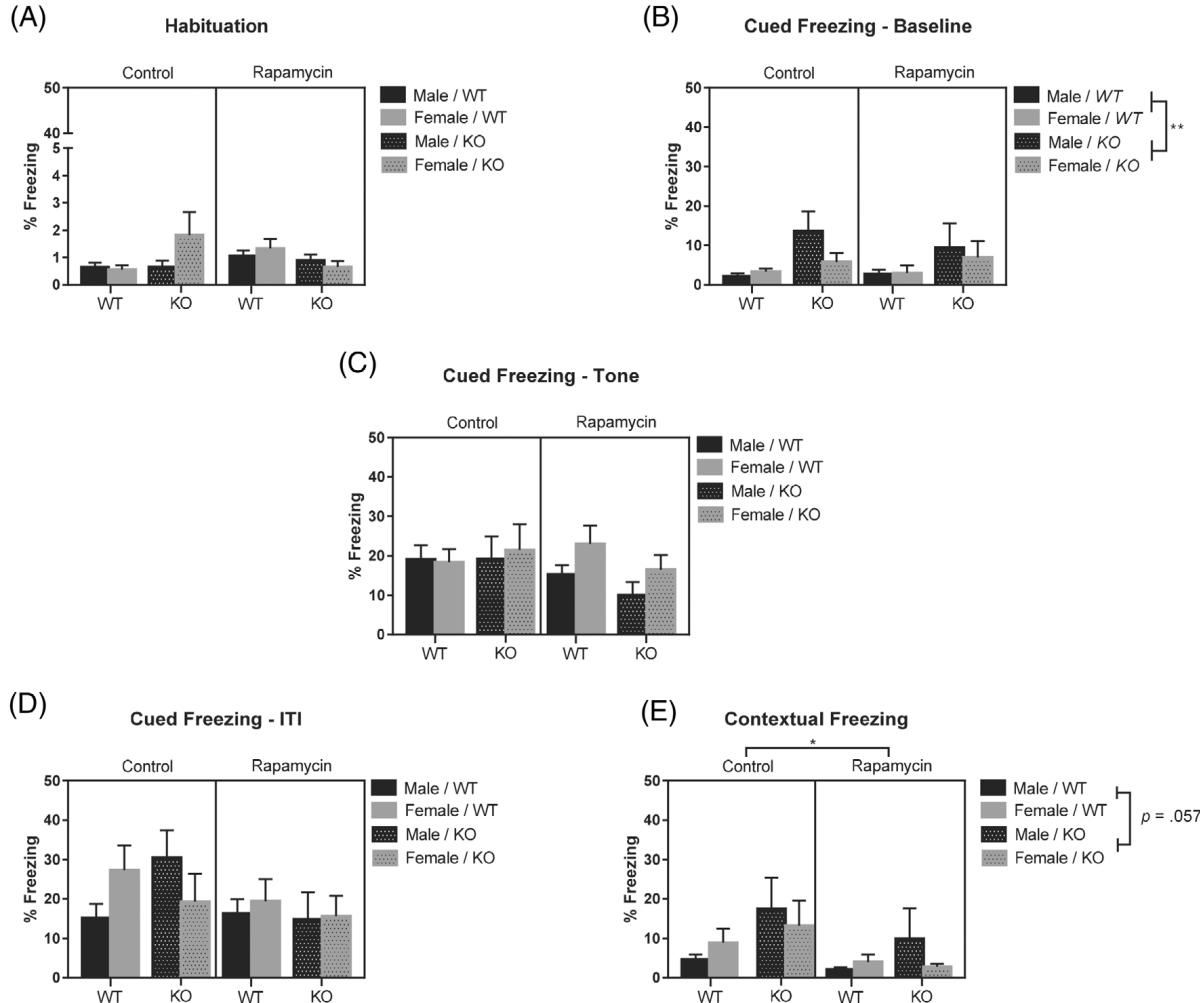
Trace fear conditioning was unaffected by rapamycin. There were no differences in activity levels during habituation. Y axis scale adjusted for visualization of data (A). Knockout (KO) mice spent more time freezing to the novel context compared with wildtype (WT) mice (B) but not during the tone (C) or inter‐trial interval (D). In the trained context, mice administered rapamycin froze less than control mice and KO mice spent marginally more time freezing compared with WT mice (E). Data are presented as mean ± SEM. *p < 0.05; **p < 0.01.

Three mice were removed from analysis because of death, seizures during training, or equipment malfunction. After removing these mice, the groups sized for the control groups were 14 female WT and 15 female KO mice, 17 male WT and 13 male KO mice. For the rapamycin treated mice there were 13 female WT and 8 female KO mice, 21 male WT and 13 male KO mice. During habituation, mice were placed in the testing chamber without the CS (white noise) or US (foot shock) to determine baseline activity levels and habituate the mice to the chamber. A three‐way ANOVA was used to compare the percent of time spent freezing in the chamber. While there was a two‐way interaction between genotype and treatment, *F*(1, 101) = 4.38, *p* < 0.05, however, after post hoc analyses there were no significant differences (Figure 6A).

Mice were assessed for cued memory performance using four presentations of the CS in an altered testing chamber 24 h after training. The within‐subjects factor, “trial,” consisted of the average percent freezing behavior during the baseline, tone and ITIs for each level of the between‐subjects factors (sex, genotype and treatment). We analyzed these factors using a repeated measures ANOVA, and analysis of the within‐subjects effects showed an interaction between genotype and trial, *F*(2, 198) = 3.77, *p* < 0.05. Pairwise comparisons of percent freezing at each trial across genotype showed that KO mice froze more than WT mice at baseline, *p* < 0.01 (Figure 6B), but not during the tone (Figure 6C), or ITI (Figure 6D). There were no other significant findings. There were also no overall differences in freezing as calculated in the between‐subjects effects.

Twenty‐four hours after the cued memory test, we placed mice in the original testing chamber to evaluate contextual fear memory. A three‐way ANOVA analyzing the differences in percent freezing during the time each mouse was in the chamber showed a main effect of treatment, *F*(1, 98) = 4.26, *p* < 0.05 but not sex. Control mice froze more than rapamycin treated mice (Figure 6E). There was also a marginal main effect of genotype, *F*(1, 98) = 3.72, *p* = 0.057. KO mice showed a marginal increase in freezing compared with WT mice (Figure 6E).

## 4 DISCUSSION

Approximately 25% of individuals with *PTEN* mutations develop autism and are at a higher risk of developing other conditions commonly found comorbid with ASD.^11^ These include macrocephaly, epilepsy and cognitive deficits which can be detrimental to quality of life.^35^, ^36^, ^37^, ^38^ NS‐*Pten* KO mice are characterized by *Pten* deletion from pyramidal cortical cells and granule cells of the hippocampus and cerebellum.^22^ These mice exhibit hyperactive mTOR signaling as a result of this mutation and develop epilepsy, macrocephaly, autistic‐like behaviors and cognitive impairments.^15^, ^22^, ^24^, ^25^, ^26^, ^27^, ^28^ A 2‐week treatment regimen of rapamycin was previously shown to suppress mTOR signaling in NS‐*Pten* KO mice.^19^, ^20^, ^21^ Here, we found that rapamycin induced an initial suppression of general activity in the open field, corrected stereotypies and increased sociability. Moreover, we showed that sex has no impact on the development of autistic‐like behaviors in NS‐*Pten* KO mice, nor on the attenuation of these behaviors by rapamycin in this model. We also showed that NS‐*Pten* KO mice show an aberrant enhancement in the generalization of fear memory not diminished by the rapamycin treatment regimen utilized in this study.

### 4.1 Rapamycin has no impact on anxiety‐like behavior in KO mice and generally suppresses activity in the open field

Both KO and WT mice showed a general suppression of activity in the open field after rapamycin treatment. They traveled a shorter distance along the perimeter and overall, and performed rearing and circling behaviors less often. In other rodent models of ASD associated with neuronal hyperactive mTOR signaling, rapamycin had little or no effect on activity in the open field.^18^, ^39^, ^40^, ^41^ For example, using an enolase promoter driven *Pten* KO model, others have found rapamycin had no impact on total distance traveled in the open field in WT or KO mice.^18^ However, these previous studies used different doses, treatment regimens and background strains. As we did not find a general suppression of activity in any of the subsequent tasks, it is possible that the hypoactivity observed in the open field was only an initial behavioral response to rapamycin treatment. Although the regimen used here suppresses neuronal hypertrophy and seizures in this model,^19^, ^20^, ^21^ it may also initially reduce general activity. As in our previous studies, we found KO mice exhibit a small but significant increase in time spent in the open arms of the elevated plus maze, indicative of reduced anxiety‐like behavior.^15^, ^26^ Although we found rapamycin had no effect on anxiety‐like behavior in the elevated plus maze in either WT or KO mice, we observed a reduction in exploratory behavior in the open field of treated WT but not treated KO mice. As the rapamycin induced reduction in activity is associated with less exploratory behavior in treated WT but not treated KO mice, this side effect may only be found in those with typical, rather than hyperactive mTOR activity.

### 4.2 Rapamycin corrects stereotypic behavior

Stereotypies are compulsive behaviors that are important to the survival and reproductive success of an animal. Mice bury foreign objects that are potentially harmful, investigate small holes that may contain food, and groom themselves to keep clean. In cases of disease or stress, an aberrant enhancement or suppression of these behaviors can be observed.^42^ Although mice with pre‐mitotic deletion do not exhibit impairments in stereotypic behavior, NS‐*Pten* KO mice were previously shown to show an overall impairment in stereotypic behavior compared with WT mice across a variety of tasks, including the hole‐board and marble burying test, as well as grooming behavior in an open field.^15^ In the current study, we found KO mice exhibit a reduction in marble burying behavior and an increase in grooming behavior in the open field. Rapamycin administration corrected the performance of both behaviors, reducing the enhanced grooming behavior in the open field and increasing marble burying performance.

It is important to consider that rapamycin may increase anxiety like behavior,^43^, ^44^ and that marble burying is enhanced when mice are under duress.^42^ While untreated KO mice were less active and showed increased grooming behavior in the open field in the current study, rapamycin did not result in anxiety‐like behavior in the elevated plus maze or suppress exploratory behavior in the open field task in KO mice. Therefore, it is unlikely that rapamycin‐induced anxiety is the cause of the increases in marble burying observed here. KO mice have been previously shown to show impaired motor capabilities.^22^, ^25^ While marble burying may generally be an effective measure of stereotypic behavior, mice with altered motor activity can confound the outcome of this task.^42^ As the stereotypic behavior produced during marble burying in the treated KO mice is improved by rapamycin, it is possible that motor capabilities may be improved and allow for enhanced performance during the marble burying test. Future studies should further examine the possibility that rapamycin treatment may improve the motor capabilities of KO mice.

### 4.3 Rapamycin increases sociability

Previous studies have showed NS‐*Pten* KO mice exhibit reduced social behavior in both the three‐chamber social task and the social partition task, wherein KO mice spent less time than WT mice investigating and interacting with a novel mouse.^15^ We were able to reproduce this social impairment also using the three‐chamber social interaction task and show that KO mice spend less time in the chamber and at the cup of a novel conspecific. Although the effect of rapamycin on social behavior was not genotype specific, treatment may have improved the social deficits in KO mice. While treated mice did not spend more time at the cup housing the novel mouse, they did spend more time in the novel mouse chamber compared with untreated mice. It is possible that the effects we observed here may have been more robust if social behavior was tested immediately after treatment. In other KO animal models of hyperactive mTOR signaling, such as the NSe‐*Pten*,^18^ *Tsc*,^45^ and *Cntnap2* ^40^ KO strains, impairments in social behavior can be corrected by inhibiting mTOR signaling with rapamycin. Our data supports mounting evidence that suppression of mTOR hyperactivity via rapamycin may improve social behavior deficits that occur secondary to enhanced mTOR signaling. We add to this body of literature by demonstrating that the social deficits that arise from pre‐mitotic deletion of *Pten* may also be reversed by rapamycin.

### 4.4 Rapamycin impairs contextual fear memory

We found rapamycin had no impact on freezing in a novel context or to the auditory cue but reduced freezing in both WT and KO mice in the trained context. Contextual fear memories have been shown to rely on proper mTOR signaling in the dorsal hippocampus^46^, ^47^, ^48^ and a single intraperitoneal injection of rapamycin can impair contextual memory up to 3 weeks later.^46^, ^48^ Although rapamycin reduced freezing in the trained context, in the current study we found trace fear conditioning still resulted in the acquisition of contextual fear memories. Memory impairments associated with systemic rapamycin typically occurs with acute doses 2–4 times larger than that administered here.^46^, ^48^ When rapamycin is administered over repeated sessions at smaller doses, memory may be less effected. These and our data collectively suggest the memory deficits associated with rapamycin are minor at the 10 mg/kg dose used here.

### 4.5 KO mice show an enhanced generalization of fear memory

KO mice exhibited an increase in baseline freezing in the novel context after fear training, demonstrating heightened fear memory generalization. Behavioral test batteries increase handling and subject mice to multiple novel test environments, both of which can alter behavioral test performance.^49^ Although we ordered the behavioral battery in a way that reduces the impact of multiple testing, it may still have affected the outcome of tests associated with fear‐ and anxiety‐like behavior. The enolase driven NSe‐*Pten* KO mice, which exhibit post‐mitotic *Pten* deletion, were reported to be easily agitated, aggressive to handling and sensitive to medication administration.^16^, ^18^ It is possible that our model may also exhibit a lower threshold to handling and multiple testing. In our previous analysis of fear memory in this model, KO mice were tested once, did not undergo an entire behavioral battery and were not handled for medication administration.^24^ Our results support the literature suggesting *Pten* deletion in mice may promote an increased sensitivity to stress.^16^, ^18^, ^50^ Additionally, anxiety disorders are a common comorbidity in ASD^51^ and occur in ~20% of those with ASD and intellectual disabilities.^52^ Individuals with ASD can become agitated when their routines are disturbed and this may result in an increase in the severity of perseverative behavior.^51^ Future analyses should determine the behavioral consequences of hyperarousal within the context of perseverative behavior in this model.

## 5 CONCLUSION

Here we show that mTOR pathway inhibition with a 2‐week course of rapamycin at 10 mg/kg starting at ~4 weeks of age increases sociability and corrects stereotypic behavior in NS‐*Pten* KO mice. As this regimen of rapamycin reverses neuronal hypertrophy and impedes the progression of seizures and subsequent mossy fiber sprouting for ~3 weeks in this model^19^, ^21^ but cannot reverse the aberrant migration of *Pten* null neurons,^53^ these data provide evidence suggesting that hypertrophy and seizures may contribute to the development of autistic‐like behaviors in NS‐*Pten* KO mice. However, mTOR also regulates protein and lipid synthesis underlying a variety of neuronal mechanisms, such as dendritic arborization, synaptic plasticity and excitability, that were not examined here.^54^, ^55^, ^56^ PTEN mutations also lead to a loss of inhibitory interneurons^57^ and increased neuroinflammation,^20^, ^50^ both of which have been associated with ASD. Thus, it is also possible that NS‐*Pten* deletion underlies both behavioral deficits, as well as other mechanisms governing neuronal structure and function. Although ASD can be diagnosed as early as 18 months, the average age at diagnosis is ~5.4–7.4 years old.^58^ Data from other rodent models of hyperactive mTOR induced epilepsy and ASD‐like behavior suggest that the earlier treatment can begin, the more likely ASD‐like behaviors can be affected.^18^ Here we also show that an acute treatment provided during the juvenile period may impact behavior in early adulthood, identifying the juvenile period as a potential developmental window of treatment. Further work, however, is needed to understand the mechanisms underlying how NS‐*Pten* KO contributes to behavioral dysfunction and how this is impacted by rapamycin treatment. Our findings expand our understanding of the ASD‐like behavioral phenotype expressed in NS‐*Pten* KO mice, open new avenues for future behavioral research and show potential promise for the treatment of ASD in those PTEN mutations.

## CONFLICT OF INTEREST STATEMENT

The authors report no conflicts or potential conflicts of interest.

## Data Availability

Data available on request from the authors.
